# The nutritional roles of zinc for immune system and COVID-19 patients

**DOI:** 10.3389/fnut.2024.1385591

**Published:** 2024-04-19

**Authors:** Di Jin, Xinran Wei, Yunyi He, Luying Zhong, Huijie Lu, Jiaxin Lan, Yuting Wei, Zheng Liu, Hongbo Liu

**Affiliations:** ^1^Guangxi Key Laboratory of Metabolic Reprogramming and Intelligent Medical Engineering for Chronic Diseases, Department of Laboratory Medicine, Guangxi Clinical Research Center for Diabetes and Metabolic Diseases, The Second Affiliated Hospital of Guilin Medical University, Guilin, China; ^2^College of Medical Laboratory Science, Guilin Medical University, Guilin, China

**Keywords:** zinc, nutrition, immune cells, interleukin, COVID-19

## Abstract

Zinc (Zn) is a vital micronutrient that strengthens the immune system, aids cellular activities, and treats infectious diseases. A deficiency in Zn can lead to an imbalance in the immune system. This imbalance is particularly evident in severe deficiency cases, where there is a high susceptibility to various viral infections, including COVID-19 caused by SARS-CoV-2. This review article examines the nutritional roles of Zn in human health, the maintenance of Zn concentration, and Zn uptake. As Zn is an essential trace element that plays a critical role in the immune system and is necessary for immune cell function and cell signaling, the roles of Zn in the human immune system, immune cells, interleukins, and its role in SARS-CoV-2 infection are further discussed. In summary, this review paper encapsulates the nutritional role of Zn in the human immune system, with the hope of providing specific insights into Zn research.

## 1 Introduction

The nutritional importance of trace metal has been known for a long time, but in the last decades its importance in immune modulation has arisen. The nutritional roles of trace metals are significant, not only as constituent components of many metal proteins and metalloenzymes, but also as essential micronutrients (1). They are required for various body functions and the well-being of the immune system (2). Balanced levels of trace metals are essential for maintaining immunity and are crucial in the prevention and management of viral infections (3). Several trace metals, such as Zn, are essential for the normal functioning of the immune system and immune-cell homeostasis. Increasing the Zn concentration can efficiently inhibit virus replication in host cells, thus exhibiting antiviral activity (4). Zn deficiency in humans is an emerging global health issue. Deficiencies of Zn often coexist with infectious diseases and exhibit complex interactions (5). Furthermore, Zn supplementation has shown a positive impact on enhancing immunity in viral infections (6). This trace metal has immunomodulatory functions, influencing the susceptibility, course, and outcome of a variety of viral infections (7). Given the current COVID-19 pandemic, where no effective preventive or curative medicine is available, a healthy immune system is one of our most important defenses.

In recent years, serum trace metal levels have been frequently reported as reliable markers for diagnosing various infectious diseases, such as COVID-19 (8). An exuberant innate immunoinflammatory response is a hallmark of severe COVID-19 with the cytokine storm, hyperinflammation, and multiorgan failure, which are correlated with elevated blood levels of proinflammatory cytokines and chemokines, e.g., IL-1β, IL-2, IL-4, IL-6, IL-8, IL-10, IL-17, IFN-γ, and TNF-α (9). SARS-CoV-2 is a pathogenic coronavirus of COVID-19, which caused a pandemic of acute respiratory disease (10). The cellular anti-SARS-CoV-2 response starts from NK cells through cytokine production. Subsequently, T cells destroy infected cells, whereas B cells to produce antibodies (11). The structural or non-structural SARS-CoV-2 proteins can elicit a host immune response. Among them, the S protein of SARS-CoV-2 contains a receptor-binding domain that serves as the critical target for antiviral compounds. B cells elicit an early response against the nucleocapsid protein of SARS-CoV-2, while antibodies against S protein could be detected after 4–8 days from the appearance of initial symptoms (12). This article reviews the nutritional roles of Zn on the immune system and SARS-CoV-2 infections, highlighting the importance of this trace metal in not only optimizing the immune response to infections but also in understanding its role in COVID-19 therapeutic approaches.

## 2 Zn in human health

### 2.1 The nutritional roles of Zn in human body

**Figure 1:**
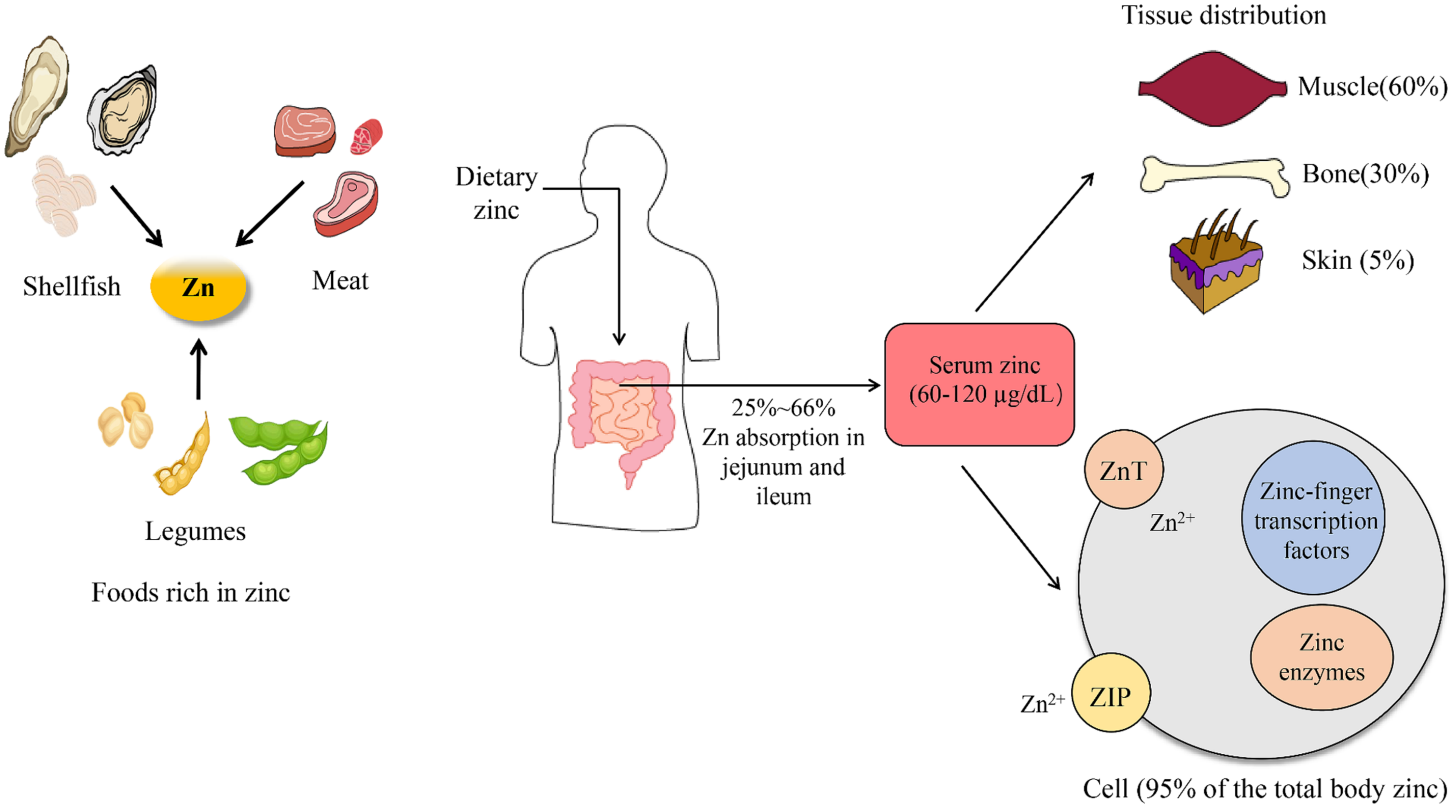
Zinc uptake and absorption.

In the adult human body, approximately 2–3 g of Zn is present (13). The reference range for serum Zn concentration in healthy individuals is between 60 and 120 μg/dL, although there may be slight regional or national variations (14, 15). For example, the reference range for Zn concentration in the human body in Bangladesh is 60–120 μg/dL (16), in Japan is ≥80 μg/dL (17), and in U.S. is 80–120 μg/dL (18). In medical testing, serum Zn levels below 60 μg/dL are indicative of inadequate Zn status (19). This inadequate Zn status is associated with a wide variety of systemic disorders, including cardiovascular impairment, musculoskeletal dysfunctions, and oromaxillary diseases (20). The human body requires daily adequate amounts of Zn, which it obtains from food or supplements. The jejunum and ileum are the primary organs for Zn absorption (Figure 1). Approximately 25–66% of consumed Zn is absorbed from the jejunum and ileum, and then distributed throughout the body (in tissues, cells, and fluids). The distribution of Zn varies across different tissues within the human body (21). The highest content of Zn is found in skeletal muscles (57%), bones (29%), and skin (6%) (22). Over 95% of total body Zn is present in the intracellular compartments, bound to intracellular proteins and cell membranes (Figure 1) (23).

Zn plays a crucial role in various processes within the human body, functioning in two states: Zn-finger transcription factors and Zn enzymes. It is involved in the synthesis of numerous proteins, as all Zn-finger transcription factors require Zn for gene expression regulation (24). Additionally, Zn serves as an activator for more than 300 Zn enzymes and participates in all biochemical reactions dependent on these enzymes (25). These reactions encompass growth, development, neurological behavior, immune system functionality, catalytic functions, acceleration of chemical reactions, modulation of neuronal communication, maintenance of cell membrane integrity and tissue balance, protein and DNA synthesis, wound healing, and cell signaling and division (26, 27). Zn also possesses antioxidant and anti-inflammatory properties that prevent damage to cells (28). Clinically, Zn supplementation is used for treating several infectious diseases, such as diarrhea, malaria, and COVID-19 (29). Of particular interest to many researchers is the fact that Zn is a nutrient required for maintaining a healthy sense of taste and smell (30).

### 2.2 Maintenance of Zn concentration in human body

Zn homeostasis is primarily maintained through the gastrointestinal system, which manages the absorption of exogenous Zn and the excretion of endogenous Zn (31). The primary site for the absorption of exogenous Zn in humans is believed to be the proximal small bowel, specifically the distal duodenum or proximal jejunum (32). The absorption of Zn involves several steps. In the stomach, the strong acidic pH liberates Zn from salts, converting it into an ionic form. These Zn ions then travel to the small intestine. At the intestinal brush border membrane, Zn is absorbed from the lumen into the enterocytes. The Zn is then excreted at the basolateral side of the enterocytes, releasing Zn ions into the portal blood, which distributes the ions throughout the body (33, 34). To maintain homeostatic balance in the body, Zn is excreted or eliminated through the kidneys, skin, and intestines when necessary (35). After circulating in the body, Zn is excreted into the small intestinal lumen along with pancreatic secretions and bile (36). Most of the Zn is reabsorbed by the duodenum and proximal jejunum, and the remaining portion is excreted in the feces (37). A significant source of fecal Zn is the pancreatic and biliary secretion of Zn-containing enzymes (38). In addition to inadequate consumption of Zn, reduced intestinal absorption is a common cause of Zn inadequacy (39). The amount of Zn excreted in the feces can vary between 0.8 and 2.7 mg per day. The daily urinary Zn excretion is approximately 0.5 mg.

Certain food-derived compounds, such as Phytic acid and Casein, can interfere with the absorption of Zn. Phytic acid, a unique natural substance found in plant seeds (present in cereals, corn, and rice), can bind to certain dietary minerals including Iron, Zn, Manganese, and to a lesser extent, Calcium (40). This binding can slow their absorption from consumed meals (41). Casein, which makes up about 80% of the total protein in cow’s milk, also exerts a modest inhibitory effect on Zn absorption (42, 43). Therefore, the consumption of cow’s milk can potentially lead to Zn deficiency. The binding of Zn to casein is pH-dependent. At slightly alkaline pH, 1 mg of casein binds 8.4 μg of Zn (44). In addition, some trace elements also affect the absorption of Zn. For instance, iron supplementation can negatively influence Zn absorption. Supplements containing 25 mg of iron or more can reduce Zn absorption and plasma Zn concentrations. This is because Zn and Iron, both positively charged ions, compete with each other for intestinal absorption (45).

Copper and Zn are antagonists, competing for the GABA (A) receptor sites in the body (46). This implies that an excess of either mineral can cause and mask a deficiency of the other. High dietary Zn intakes depress copper absorption, increase copper sequestration in the mucosal cell bound to metallothioneins, and increase fecal excretion of copper (46). Conversely, Zn-deficient animals have shown increased copper absorption, and high dietary copper can depress Zn absorption (47). According to research, the ideal Zn to copper ratio is between 8:1 and 15:1 (48). Maintaining this ratio is crucial for good health and various physiological functions. An excess of copper, coupled with a lack of Zn, may predispose an individual to oxidative stress and trigger the inflammatory process (49). Disruption of this balance can lead to several health conditions. For instance, copper toxicity can cause symptoms such as diarrhea, headaches, and in severe cases, kidney failure (50). On the other hand, Zn deficiency can result in growth retardation, hypogonadism, immune dysfunction, and cognitive impairment (51).

Zn plays a crucial role in the absorption of certain vitamins. It facilitates the absorption of vitamin A, vitamin E, and folate1 (52). Zn is a component of the retinol-binding protein, which is necessary for transporting vitamin A in the blood (53). Additionally, Zn is required for the enzyme that converts retinol (vitamin A) to retina (54). The exact mechanism by which a deficiency in Zn affects the absorption of vitamin E and folate is not entirely clear. However, it is known to involve the loss of Zn enzyme function. Special attention should be given to the balance of intracellular and extracellular Zn concentrations. Cellular Zn homeostasis is primarily maintained by the Zn importers family, ZnT (SLC30), which allows Zn to accumulate in the cytosol, and by the Zn exporters’ family, ZIP (SLC39) (55). The main controllers of intracellular Zn concentration are the ZnT importers, ZIP exporters, and intracellular binding proteins known as metallothioneins (55).

### 2.3 Zn uptake in human

The recommended daily intake of Zn varies across countries. The World Health Organization (WHO) recommends a dietary Zn intake of 9.8 mg/day for women and 14 mg/day for men, both aged between 19 and 65 years (56). In the U.S., the current recommended daily value (DV) for Zn is 8 mg for women and 11 mg for adult men (57). The requirements increase slightly during pregnancy and lactation. In the UK, the reference nutrient intake is 4–7 mg/d for females and 5.5–9.5 mg/d for males (Food Standards Agency) (58). In Australia, the current Recommended Dietary Intake (RDI) for Zn is 14 mg/day for men and 8 mg/day for women (19+ years), with an upper limit of 40 mg/day (59). In Germany, the recommended intake values for Zn range from 7 mg/day to 16 mg/day for adults, including pregnant and lactating women, depending on sex and dietary phytate intake (60). The Ministry of Health, Labor and Welfare in Japan recommends a standard Zn intake of 10 mg per day for men and 8 mg per day for women (61). According to the Chinese dietary guidelines, the recommended daily Zn intake for adults over 18 years old is 7.5 mg for women and 12.5 mg for men, with tolerable upper intake levels of 40 mg (62).

A Zn deficiency can lead to small stature, mild anemia, and impaired wound healing. Good sources of Zn are meats, whole grains, and legumes. The Recommended Dietary Allowance (RDA) is 8 mg/d for women and 11 mg/d for adult men (63). The amount of Zn provided by the U.S. food supply has varied between 11 and 13 mg/day/person since the beginning of the 20 century. Currently, it is estimated that the U.S. food supply provides 12.3 mg Zn per person per day (64). While red meat is a particularly good source, all kinds of meat, including beef, lamb, and pork, contain Zn (65). According to the information provided by U.S. Department of Agriculture,^1^ a 100-gram (3.5-ounce) serving of raw ground beef contains 4.79 mg of Zn. This is approximately 44% of the DV for males and 60% of the DV for females. Shellfish are healthy, low-calorie sources of Zn. Oysters, in particular, contain high amounts, with six medium oysters providing 33 mg, or 300% of the DV for males and 413% of the DV for females. Legumes, such as chickpeas, lentils, and beans, contain substantial amounts of Zn. Table 1 lists the 10 foods with the highest Zn content.

**Table 1:** The top 10 foods with the highest zinc content.

Description	Serving size	Zn per size (mg)	Zn per 100 g (mg)
Egg, yolk, dried	1 oz	2.02	7.11
Seeds, pumpkin seeds (pepitas), raw	100 g	6.34	6.34
Nuts, pine nuts, raw	100 g	5.71	5.71
Beef, flank, steak, boneless, choice, raw	100 g	5.56	5.56
Egg, whole, dried	5 g	0.25	5.02
Flaxseed, ground	100 g	4.74	4.74
Cheese, parmesan, grated, refrigerated	100 g	4.62	4.62
Flour, soy, defatted	100 g	4.44	4.44
Lentils, dry	100 g	3.86	3.86
Beans, dry, medium red (0% moisture)	100 g	3.82	3.82

## 3 Zn and immunity

### 3.1 Zn play a central role in the immune system

Zn is a member of the transition metals family, characterized by low ionization energies and a broad spectrum of oxidation states, or positively charged forms (66). It plays a significant role in regulating cytokine expression and suppressing inflammation. Zn is necessary for the activation of antioxidant enzymes that scavenge reactive oxygen species (ROS), thereby reducing oxidative stress (67). Dysregulated Zn homeostasis can impair overall immune function, leading to increased susceptibility to infection (68). Zn has several mechanisms for combating pathogens. In the body, Zn generally exists as positively charged ions. These Zn ions act as chemo-attractants, drawing in pathogens for phagocytosis and killing them through the production of ROS (69). One such mechanism involves Zn oxide nanoparticles, which induce the generation of abundant ROS, including singlet oxygen species (1O2), within cells. An elevated level of ROS triggers an antioxidant cellular response and mitochondrial dysfunctions (70). Interestingly, researchers have discovered that Zn can ‘starve’ *Streptococcus pneumoniae* by inhibiting their uptake of manganese, a metal essential for the protein transporter that *Streptococcus pneumoniae* requires to invade and cause disease in humans (71).

Zn plays a vital role in many immune processes as a catalyst, structural element, and regulatory ion (72). The immune system, being highly proliferative, is particularly susceptible to Zn deficiency (73). Here’s a summary of how Zn impacts the immune system: (1) Zn helps control infections by moderating the immune response, thus preventing inflammation that can be damaging and even deadly (74); (2) Zn is essential for the development of a specialized type of immune cell and stimulates a critical immune organ to regenerate after damage (75); (3) Zn is necessary for immune cell function and cell signaling. A deficiency can lead to a weakened immune response (76). For instance, Zn is crucial for the normal development and function of cells mediating innate immunity, such as neutrophils and natural killer cells (77). Zn is also required for DNA synthesis, RNA transcription, cell division, and cell activation (78). In the absence of adequate levels of Zn, programmed cell death (apoptosis) is potentiated (79).

### 3.2 Zn is necessary for the normal function of the immune cells

**Figure 2:**
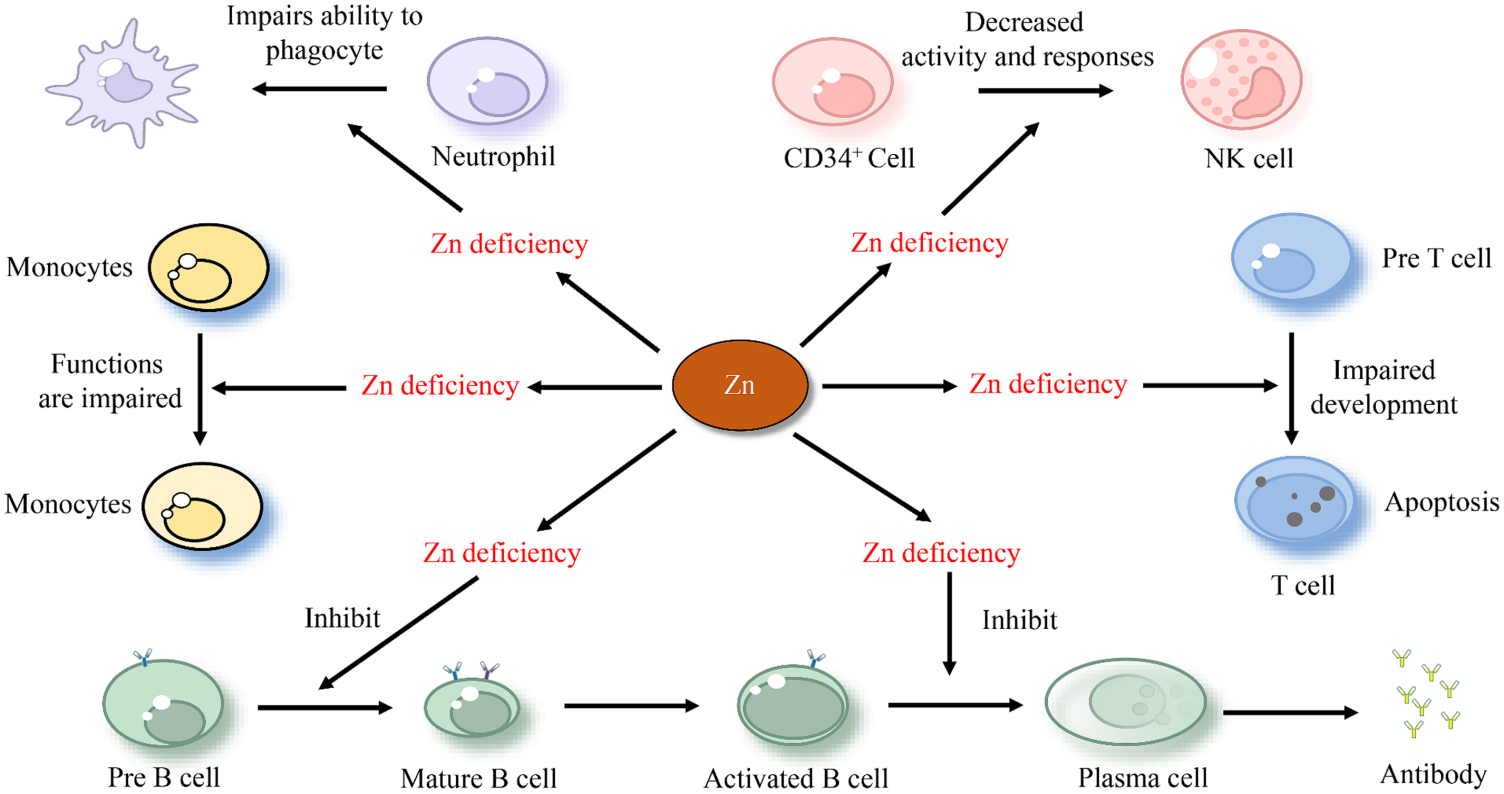
The effect of zinc on immune cells.

Zn boosts the immune system and combats viruses by enhancing the actions of immune cells such as Neutrophils, B cells, NK cells, and T cells (80). Neutrophils, a type of white blood cell, aid the body in fighting off infections by engulfing and destroying invading pathogens (81). Zn plays a pivotal role in the development and activation of neutrophils (82). A study has shown that Zn deficiency leads to increased neutrophil transmigration and impairs the ability of neutrophils to phagocytose and produce ROS (83). Research also indicates that Zn supplementation can help reduce neutrophil recruitment and activity, thereby helping to prevent lung injury (84). B cells are antigen-presenting cells that produce antibodies and cytokines, represent immunological memory, and even appear to have regulatory and suppressing functions in inflammation (85). Significantly elevated Zn levels have been observed in activated B cells (86). Existing research confirms that Zn deficiency leads to a reduction in B cells, affecting the development of immature and pre-mature B cells and impacting antibody production (87). Low levels of cytoplasmic Zn in deficient B cells were associated with reduced cell signaling during crucial stages in B cell development (88). Zn also modulates the response of NK cells, with a decreased recognition and stimulation of their MHC-class I expression (89). Furthermore, Zn supplementation increases the differentiation of CD34+ cells toward NK cells as well as their cytotoxicity (Figure 2) (90).

T cells, a crucial type of white blood cells, play a central role in the adaptive immune response (91). The impact of Zn on T cells has been confirmed experimentally. Individuals with low Zn levels have few, if any, T cells to fight infections. This may be because Zn deficiency leads to thymic atrophy and subsequent T-cell lymphopenia (92). Moreover, Zn deficiency negatively affects the growth and function of T cells and various aspects of innate and adaptive immunity, including T cell lymphopoiesis and antibody-mediated immune defense (93). Animal studies have also corroborated this conclusion. It has been demonstrated that Zn enhances the regeneration of the thymus in mice, the organ where T cells develop, and aids in immune-cell recovery post bone marrow transplant (94). According to a study, a lack of Zn during T cell maturation results in a 50% reduction in the transition from “potential” pre-T-cells to “effective” T cells. This is associated with an increased apoptosis of pre-T-cells (Figure 2) (95). Some researchers hypothesize that Zn interacts with protein kinase C and the lymphocyte protein tyrosine kinase. The dysfunction of these two kinases, which are involved in mature T-cell activation, is a reason that Zn deficiency leads to a reduced T cell count (96). Similarly, Zn deficiency results in a decreased ratio of type 1 to type 2 T-helper cells, with reduced production of T-helper type 1 cytokines like interferon-gamma (IFNG), and compromised T-cell mediated immune defense (97). Zn supplementation has been shown to enhance immunity and effectively downregulate chronic inflammatory responses (98).

### 3.3 Zn is involved in the regulation of immune reaction

#### 3.3.1 The impact of Zn on immune responses in cell lines

One of the important functions of Zn ions is to serve as second messengers. They act as intracellular signaling molecules, capable of communicating between cells, converting extracellular signals to intracellular ones, and regulating the activation of interleukins (99). Zn can induce monocytes to produce pro-inflammatory cytokines such as interleukin-1 (IL-1), interleukin-6 (IL-6), and interleukin-8 (IL-8) (100). The IL-1 family, a group of 11 cytokines, plays a central role in the regulation of immune and inflammatory responses to infections (101). Zn has been found to reduce IL-1 dependent T cell stimulation by inhibiting the IL-1 receptor-associated kinase-1 (102). IL-1β is a major cytokine involved in monocyte activation and the activation of proinflammatory signaling pathways (103). Cytoplasmic Zn promotes IL-1β production and subsequently inhibits inflammation, depending on the transcription factor NF-κB, an inflammatory activating factor (104). However, a previous study has shown that Zn depletion in macrophages induces the activation of proteases that cleave pro-IL-1β, leading to an increased release of active IL-1β (105). These studies suggest a complex correlation between Zn and IL-1.

Interleukin-2 (IL-2), which is produced by T cells during an immune response, is essential for the growth, proliferation, and differentiation of naive T cells into effector T cells (106). The level of Zn has a positive correlation with IL-2. Zn enhances the proliferation of T cells in response to IL-2, as well as the production of IL-2 by T cells (107). A previous study evaluated the circulating cytokines and Zn status, showing that reduced circulating Zn correlates with increased levels of IL-6 and IL-8 (108). The proliferative response of T and B lymphocytes following IL-6 and IL-8 stimulation increases in Zn deficiency, while it adversely influences IL-4 signaling, leading to an impairment of the immune system (109). Research has found that Zn deficiency decreases the production of TH1 cytokines (IFN-γ, IL-2, and TNF-α), whereas the TH2 response (IL-4, IL-6, and IL-10) is less affected. This results in an imbalance between TH1 and TH2 subsets, which is restored when Zn reaches physiological levels (110). The proinflammatory TH17 cells are also negatively affected by Zn deficiency. The development of TH17 cells is critically controlled by IL-6-induced STAT3 activation during chronic inflammation (111). Zn suppresses TH17 development by attenuating this activation (112).

#### 3.3.2 Impact of Zn on immune responses in animal and human studies

The effects of Zn on animals are diverse and significant. One study conducted on swine discovered that dietary supplementation of Zn in an organic form influenced the expression of inflammatory molecules within the intestine. This organic form of Zn resulted in a reduced level of the pro-inflammatory cytokine IL-18. This finding suggests that organic Zn sources could enhance the health and immune response of animals under stress by altering gene expression in the intestine (113). Another study explored the impact of Zn deficiency in rats. The researchers found that a lack of Zn exacerbated the inflammatory response, leading to hemolytic anemia and splenomegaly. Notably, the administration of IL-4 and Zn supplementation were found to reverse the hemolytic anemia and splenomegaly induced by Zn deficiency (114). In a separate study using a mouse model of allergic inflammation, it was found that Zn exhibited anti-inflammatory effects (115).

Zn deficiency has a broad impact on human health, leading to dysfunctions in both innate and adaptive immunity. This can result in diminished lytic activity, impaired signaling, altered cytokine production, and reduced proliferation. Consequently, individuals deficient in Zn are more susceptible to infectious diseases (72). Zn supplementation in the elderly has been found to decrease the incidence of infections, reduce oxidative stress, and lower the generation of inflammatory cytokines (116). A sensitivity analysis revealed that Zn supplementation decreases IL-6 levels and increases IL-2 levels (117). In an experimental model of human Zn deficiency, there was a reported decrease in thymulin activity in Th1 cells, a reduction in IL-2 and IFN-gamma genes, and diminished activity of natural killer cells (NK) and T cytotoxic T cells (118). These findings underscore the vital role of Zn in regulating interleukin expression and immune function. However, the exact mechanisms and pathways involved are complex and may vary depending on the specific context and conditions.

## 4 The potential role of Zn in preventing COVID-19

### 4.1 Clinical study of Zn in the treatment of COVID-19

Clinically, COVID-19 patients have shown an imbalance in Zn levels. Low Zn levels appear to be common in these patients (119). In a previous study, all COVID-19 patients (33 out of 33) were found to be Zn deficient, with a mean serum level of 6.9 ± 1.1 μmoL/L, which is well below the Zn deficiency cutoff value of 10.7 μmoL/L (120). This Zn deficiency observed in COVID-19 patients may be an acute phase reaction to SARS-CoV-2 infection (121). Compared to other interventions, those who received a combination of Zn and vitamin C were found to mount a greater antibody response. This suggests that oral Zn and vitamin C treatment could stimulate antibody production following SARS-CoV-2 infection (122). Zn therapy is considered logical in COVID-19 due to the inhibitory effect of Zn on viral replication (123). A study found that the combination of doxycycline and Zn has a protective effect in COVID-19 patients (124). Interestingly, Zn therapy plays a significant role in shortening the duration of smell recovery in these patients (125). It’s important to note that administering high-dose Zn appears to be safe, feasible, and associated with minimal peripheral infusion site irritation in COVID-19 patients (126).

Although case studies may not hold the same universal significance as group studies, they offer specific parameters, treatment plans, and treatment outcomes, which make them quite intriguing. Elanjian et al. reported three cases of COVID-19 patients who received Zn supplementation, with experiencing hypoglycemia (127). Rosenberg et al. reported a case of copper deficiency, which was caused by Zn supplementation in the context of COVID-19. This deficiency presented very similarly to myelodysplastic syndrome (128). Farolfi et al. reported a case of a 98-year-old patient who received vitamin D and adjuvant dietary supplements (quercetin, vitamin C, Zn, and vitamin K2) at home. The patient fully recovered, suggesting that careful home assistance under strict medical supervision can be successful, even in very old subjects with comorbidities (129). Ahmed et al. reported a case of a 48-year-old Hispanic female patient with COVID-19, who presented with severe isolated thrombocytopenia. She was treated with intravenous immunoglobulin, prednisone, rituximab, vitamin C, and Zn, and achieving hemodynamic stability (130).

### 4.2 Mechanism of Zn treatment for COVID-19

Multiple pieces of evidence indicate that Zn plays a significant role in the treatment of patients with COVID-19 and a deficiency in Zn is associated with increased severity of COVID-19. However, the exact mechanism by which Zn inhibits the virus, particularly SARS-CoV-2, remains unclear. We propose the following mechanisms: (1) Zn is known to impair the replication of several RNA viruses including poliovirus, influenza virus, and SARS-CoV-2, by inhibiting the main protease, which is crucial for the virus’s replication (131); (2) Zn is an essential trace element with potent immunoregulatory and antiviral properties (132). This suggests that Zn could inhibit inflammation and alleviate oxidative stress by inducing the aforementioned interleukin. (3) Zn is used by SARS-CoV-2 as an essential step in preparation for entering and invading cells. It latches onto the exterior of potential host cells. During this process, Zn is strongly implicated in inhibiting the virus’s binding to angiotensin-converting enzyme-2 (ACE2) receptors on the cell membrane, thus mitigating the attack by those virus particles that do manage to enter host cells (133). Supplemental Zn could replenish Zn in ACE2, stabilize the ACE2 axis, and prevent disruption of the renin-angiotensin system (134). Moreover, Zn minimizes the activity of Sirtuin-1 (SIRT-1), which regulates ACE-2 expression and could potentially block virus entry (135).

## 5 Conclusion

This review article explores the multifaceted roles of Zn in immune system functions. The dynamic equilibrium of Zn in the human body is crucial for maintaining normal immune cell function and response by inducing monocytes to produce pro-inflammatory cytokines. Given its significant role in the immune system, Zn has demonstrated positive effects and potential therapeutic benefits in the treatment of COVID-19. However, current research on the function of Zn in the immune system and its therapeutic impact on viral infections remains somewhat superficial. These research primary focus on the changes in Zn concentration in virus-infected patients, the correlation between Zn and pro-inflammatory cytokines, or the role of Zn in inhibiting virus replication or preventing virus entry into cells. Zn, serving as a structural or catalytic cofactor, fulfills various biological functions through Zn enzymes or Zn finger proteins. For instance, our recent research discovered that the Zn finger protein ZBTB34 can bind to telomere DNA, regulate telomere length, and is associated with the onset of liver cancer (136, 137). The biological functions of Zn in the immune system, such as stimulating the expression of pro-inflammatory cytokine genes, are achieved through these Zn enzymes and finger proteins. Regrettably, the functions of over 300 Zn enzymes and hundreds of Zn finger proteins are not well-understood at present. Future research should prioritize the study of Zn enzymes and Zn finger proteins, which will undoubtedly unveil more biological functions of Zn in the immune system.

## Author contributions

DJ: Conceptualization, Writing – original draft. XW: Investigation, Writing – original draft. YH: Investigation, Writing – original draft. LZ: Investigation, Writing – original draft. HLu: Investigation, Writing – original draft. JL: Investigation, Writing – original draft. YW: Investigation, Writing – original draft. ZL: Conceptualization, Funding acquisition, Supervision, Writing – review & editing. HLi: Funding acquisition, Supervision, Writing – review & editing.

## Footnotes

^1^ https://fdc.nal.usda.gov/index.html
